# Atrial Fibrillation and Chronic Coronary Ischemia: A Challenging Vicious Circle

**DOI:** 10.3390/life13061370

**Published:** 2023-06-12

**Authors:** Alexandru Florinel Oancea, Raul Alexandru Jigoranu, Paula Cristina Morariu, Radu-Stefan Miftode, Bogdan Andrei Trandabat, Diana Elena Iov, Elena Cojocaru, Irina Iuliana Costache, Livia Genoveva Baroi, Daniel Vasile Timofte, Daniela Maria Tanase, Mariana Floria

**Affiliations:** 1 Department of Internal Medicine, Faculty of Medicine, University of Medicine and Pharmacy Grigore T. Popa, 700115 Iasi, Romania; oancea.alexandru-florinel@d.umfiasi.ro (A.F.O.); morariu.paula-cristina@email.umfiasi.ro (P.C.M.); radu-stefan.miftode@umfiasi.ro (R.-S.M.); trandabat.bogdan-andrei@d.umfiasi.ro (B.A.T.); diana-elena.iov@d.umfiasi.ro (D.E.I.); elena2.cojocaru@umfiasi.ro (E.C.); irina.costache@umfiasi.ro (I.I.C.); livia.baroi@umfiasi.ro (L.G.B.); daniel.timofte@umfiasi.ro (D.V.T.); floria.mariana@umfiasi.ro (M.F.); 2 Cardiology Clinic, St. Spiridon Emergency Hospital, 700115 Iasi, Romania; 3 Internal Medicine Clinic, St. Spiridon Emergency Hospital, 700115 Iasi, Romania; 4 Department of Morphofunctional Sciences—Pathology, Pediatric Hospital, 700115 Iasi, Romania; 5 Surgery Clinic, St. Spiridon Emergency Hospital, 700115 Iasi, Romania

**Keywords:** coronary ischemia, coronary artery disease, atrial fibrillation, chronic coronary syndrome, triple antithrombotic therapy

## Abstract

Atrial fibrillation, the most frequent arrhythmia in clinical practice and chronic coronary syndrome, is one of the forms of coronary ischemia to have a strong dual relationship. Atrial fibrillation may accelerate atherosclerosis and may increase oxygen consumption in the myocardium, creating a mismatch between supply and demand, thus promoting the development or worsening of coronary ischemia. Chronic coronary syndrome alters the structure and function of gap junction proteins, affecting the conduction of action potential and leading to ischemic necrosis of cardiomyocytes and their replacement with fibrous tissue, in this way sustaining the focal ectopic activity in atrial myocardium. They have many risk factors in common, such as hypertension, obesity, type 2 diabetes mellitus, and dyslipidemia. It is vital for the prognosis of patients to break this vicious circle by controlling risk factors, drug therapies, of which antithrombotic therapy may sometimes be challenging in terms of prothrombotic and bleeding risk, and interventional therapies (revascularization and catheter ablation).

## 1. Introduction

Chronic coronary syndrome (CCS) is one of the most frequent and impactful cardiovascular diseases, while atrial fibrillation (AF) is the most common cardiac arrhythmia [1,2]. Approximately 15% of patients with AF will undergo percutaneous coronary intervention for CCS, which often coexists [3]. The prevalence of both CCS and AF increases directly with age, with CCS being the leading cause of morbidity and mortality in the elderly population. Patients with AF are older and have a higher risk of stroke, which may contribute to increased mortality. Therefore, the coexistence of CCS and AF could lead to heart failure as a “final destination”, implicating a much higher risk of mortality in the elderly population [3,4].

Despite stable symptoms, coronary artery disease is a dynamic process of atherosclerotic plaque buildup and functional alterations in the coronary circulation that can be modified by lifestyle (diet, smoking, sedentarism), pharmacological therapies, and revascularization, which may lead to the stabilization or, worse, progression of the disease and evolution to an acute event [5].

Atrial fibrillation is associated with a prothrombotic status, patients with AF having an increased risk of ischemic stroke, transient ischemic attack, and systemic embolism compared with the rest of the population. CHA2DS2-VASc score [C = congestive heart failure, objective evidence of moderate to severe left ventricular dysfunction or hypertrophic cardiomyopathy, H = hypertension or on antihypertensive therapy, A = age 75 or older (doubled), D = diabetes mellitus, S = previous stroke, transient ischemic attack or systemic embolism (doubled), V = vascular disease (prior myocardial infarction, peripheral artery disease, aortic plaque, significant coronary artery lesions on angiographic evaluation), A = age 65–74 years, Sc = sex category (female)] was created to calculate the thromboembolic risk in order to decide which chronic treatment to use with anticoagulants in patients with AF [6].

The relationship between AF and CCS is dual. On the one hand, AF is able to generate an inflammatory response by mechanisms not yet fully understood (high levels of inflammatory markers have been reported in patients with AF compared with those in sinus rhythm), which will lead to the acceleration of atherosclerosis [7]. AF also increases oxygen consumption, creating a mismatch with the blood supply in myocardium, which is the key to the pathophysiological mechanism for CCS. On the other hand, CCS can promote AF by increasing focal ectopic activity in the left atrium and by affecting micro reentry formation [8].

There is a strong connection between cardiovascular diseases, such as between CCS, AF, and their main risk factors (hypertension, diabetes, obesity, dyslipidemia). In these patients, epicardial adipose tissue loses its protective role and it is implicated in the development of both CCS and AF [9].

## 2. Pathophysiology of Atrial Fibrillation

**Figure 1:**
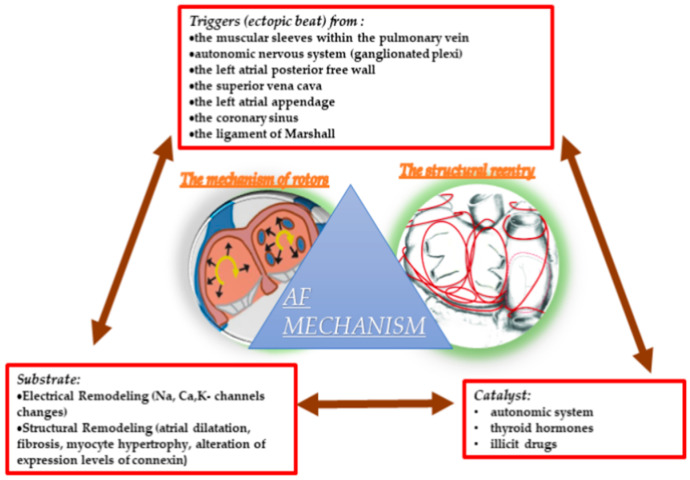
The trigger and substrate in atrial fibrillation pathogenesis.

The mechanism of AF may be associated with a concept using Coumel’s Triangle that requires a trigger for initiation, a catalyst agent, and an arrhythmogenic substrate for the perpetuation and maintenance of the trigger, as shown in Figure 1 [10].

The main source of the ectopic beats triggering paroxysmal forms of AF is represented by the muscular sleeves within the pulmonary vein ostia, these foci responding to treatment with radio-frequency ablation [11,12,13], but the autonomic nervous system, specifically innervation from ganglionated plexi in the left atrium, play a role in the generation of ectopic foci, known as early and late after depolarizations, and electrical dispersion required for AF [14]. Moreover, there is also a high prevalence of ganglionated plexi in the posterior surface of the right atrium, and their ablation may be effective in about 70% of patients with vagal paroxysmal AF [15]. However, according to *GANGLIA-AF study*, ganglionated plexi ablation (GPA) did not prevent atrial arrhythmias more than pulmonary vein isolation (PVI), and GPA had a significantly longer procedure time, but there was significantly less radiofrequency ablation and a higher reduction in the usage of antiarrhythmic drugs postablation in patients with GPA than those with PVI [16]. Finally, it seems that the addition of GP ablation to PVI is the best interventional therapeutic strategy, adjunctive GP ablation being more effective in paroxysmal AF and in patients with smaller atria [17].

For patients with persistent forms of AF, it is more likely to have non-PV triggers, such as the left atrial posterior free wall [18,19,20], the left atrial appendage [21], the superior vena cava (young patients with AF had a higher incidence of only non-PV foci, mostly located here) [22,23,24,25], the coronary sinus [26,27], and the ligament of Marshall [28,29]. For non-PV foci patients, the isolation of them was a significant factor affecting the achievement of better clinical outcomes following AF ablation [30]. Moreover, PVI extended to the entire posterior wall which may bring better results regarding long-term arrhythmia-free survival in patients with persistent AF and without inducible non-PV triggers [31].

The catalyst, which can act on both the substrate and the trigger, changing refractory periods and increasing automatic activity, is represented by the autonomic system, thyroid hormones, and other factors such as illicit drugs [10]. A recent study claims that the autonomic nervous system may mediate acute apnea-induced atrial fibrillation. In patients with obstructive sleep apnea, there is a chronic increase in parasympathetic nervous modulation and relative reduction in sympathetic modulation, and this altered autonomic function may accelerate arrhythmogenesis (paroxysmal forms of AF) and impair antiarrhythmic therapy [32].

The substrate develops because of electrical remodeling (which shortens the refractory period and duration of the atrial action potential) or structural remodeling (which prevents the propagation of the action potential by decreasing its conductivity) [33].

The electrical one consists of a complex electrical substrate and ionic mechanism changes in the atria [34]. The channels involved are used in the depolarization, repolarization, and conduction of the action potential in the atrial myocardium (calcium, sodium, potassium). Calcium overload is one of the main causes of electrical remodeling; it is a ubiquitous intracellular signal mediator, which has been shown to have a negative feedback effect on L-type calcium channel activity with a decrease in the plateau phase of the action potential, sustaining that during atrial fibrillation, its high levels may shorten the duration of the action potential [35]. The role of sodium in electrical remodeling is controversial; there are studies that sustain there is no change in the Na+ current density nor in the expression of the Na+-channel subunits at the mRNA level in cardiomyocytes from patients with AF, and others that claim an association of familial AF and LQT-3 as a consequence of a single mutation (Y1795C) in the SCN5A gene [36,37]. Potassium channels mutations have also demonstrated a strong familial penetrance for AF, some of them, such as s Ito, Iku, and IK1, being involved in electrical remodeling [36,38].

Structural remodeling occurs on a much shorter time scale than electrical remodeling. It consists of conformational changes in the left atrium, such as alterations in the expression levels of connexin (the protein comprising the gap junctions that couple cells), myocyte hypertrophy, myocardial fibrosis, and, finally, the atrial dilatation [39,40,41]. On the one hand, fibrosis and decreased connexin levels prevent action potential propagation through myocytes, and on the other hand, atrial dilatation and cellular hypertrophy increase the atrial activation time [33,42].

The mechanism of reentry in AF is still controversial with two dominant hypotheses: micro or macro structural reentry and fixed or moving functional reentrant rotors. It is important that the propagating wave front must complete one circle movement in a time period long enough for the atrial tissue within that circuit to recover excitability, making the perpetuation of functional reentry possible [43].

The reentry occurs in the presence of unidirectional block and of slow conduction that makes the wavelength shorter than the length of the circuit, these conditions being favorized by the fibrosis in the atria. Around these fibrotic areas, multiple waves propagate randomly, and they may give birth to new daughter wavelets. In most cases, the reentrant drivers are located in the pulmonary vein antrum and surrounding structures, around the left atrial appendage, and around interatrial septum. There is also micro-anatomic reentry localized in the right atrium, within 1–2 mm area, that may appear in the presence of non-uniform anisotropic conduction and micro-fibrosis of the pectinate bundles. The left atrium, where it is an area with abrupt changes in the myocardial fiber orientation, localized at the junction between the posterior wall and the left atrial roof, may be also a source of micro-reentry [44,45,46,47].

The mechanism of “rotors” or spiral waves In human AF is still controversial. Rotors, also known as functional reentry, consists of an unexcited core with a potential for action surrounding it. In contrast to anatomical reentry (fibrotic tissue), rotors have their core excitable. They may be initiated, including by a sinus wave, due to a wavefront break. Electrical remodeling associated with reduced refractoriness supports multiple wavelet reentry and thus the activity of “rotors” [48,49].

## 3. Pathophysiology of Chronic Coronary Ischemia

Coronary ischemia, whether acute or chronic, is the leading cause of mortality worldwide. In 2019, the European Society of Cardiology (ESC) defined “Chronic coronary syndrome” instead of “stable coronary artery disease (CAD)”. This syndrome includes six clinical scenarios: stable angina, new onset of HF in patients with suspected CAD, subjects with recent revascularization or with stabilized symptoms < 1 year after an ACS (acute coronary syndrome), patients > 1 year after initial diagnosis or revascularization, symptomatic patients with suspected vasospastic or microvascular disease, and asymptomatic ones in whom CAD is confirmed [50].

**Figure 2:**
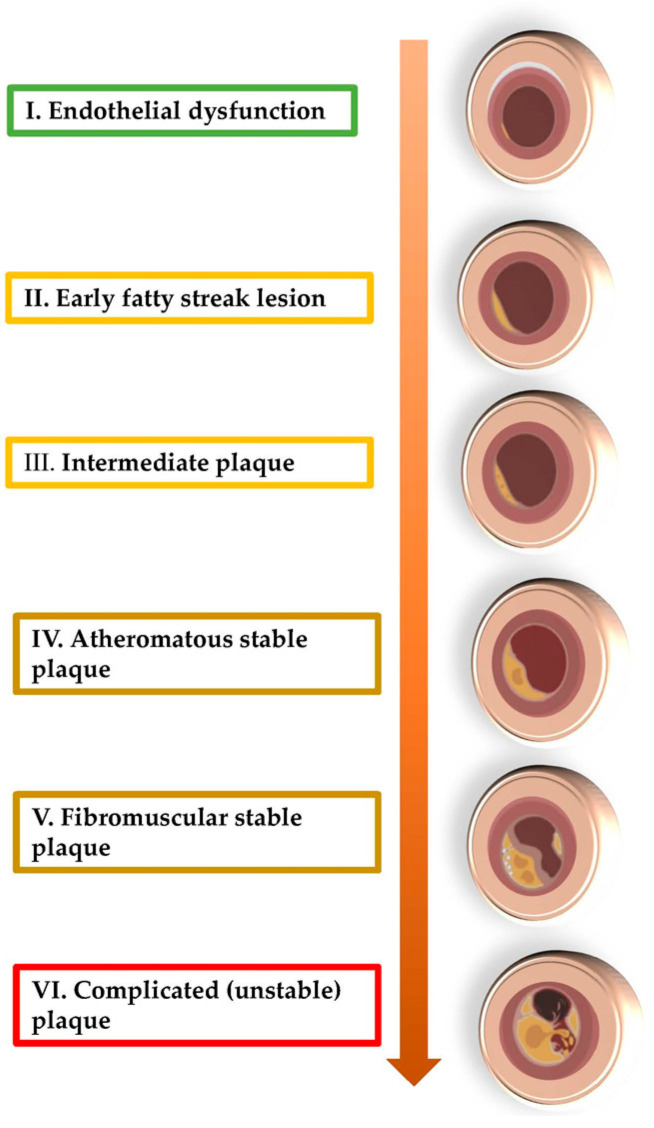
The evolution of atherosclerotic plaque.

The main cause of chronic coronary ischemia is atherosclerosis, an unavoidable process that starts from intrauterine life. The first detectable change in atherosclerotic plaque is endothelial dysfunction. Due to this, monocytes will be capable of diapedesis to reach into the subendothelial tissue, where they will transform into macrophages. These cells will phagocytize lipoproteins and LDL-cholesterol, to become foam cells, which correspond to the early fatty streak lesions. Then, the muscle cell division is stimulated by chemokines and growth factors elaborated by activated endothelium and macrophages. In this way, after collagen synthesis, the plaque will become now a fibromuscular one, while a fibrous cap will cover a lipid-rich and necrotic core, which is formed by oxidized lipoproteins and cholesterol molecules; in the periphery of the plaque, a rich population of leukocytes such as macrophages, dendritic cells, and T-lymphocytes will develop. These cells will amplify the instability of the plaque, which may lead to a fatal transition from chronic to acute events because the rupture of the plaque will trigger the coagulation cascade and, finally, the thrombus formation (Figure 2). In general, a reduction in luminal diameter of more than 70% is required to significantly restrict blood flow, generating the specific symptoms of chronic ischemia [51,52].

Regarding others causes of CCS, in which there is no evidence of stenosis in coronary tree, microvascular territory, the spasm, or myocardial bridging may be incriminated. The microvascular vessels, which may be affected anatomically (atherosclerosis) or functionally (exaggerated vasomotor tone), are one of the causes of INOCA (ischemia with non-obstructed coronary artery disease) [53].

## 4. From Coronary Ischemia to Atrial Fibrillation

One of the many risk factors in the development of AF, despite hypertension, diabetes, and obesity is CAD, as shown in the Framingham Study, which suggested that CCS predisposed to AF: it doubled the risk among men (for all types of AF) and quadrupled it among women (only for paroxysmal AF) [54,55].

A possible pathophysiological explanation may be given by the stenosis of the vascular tree irrigating the sinoatrial node, which is a key in the perpetuation of arrhythmias, due to the difficulty of maintaining sinus rhythm in these patients. A recent study, involving patients with CAD demonstrated by cardiac catheterization, affecting especially the sinoatrial nodal artery, the atrioventricular nodal artery, and right intermediate atrial artery, as well as the entire right coronary, shows that the odds of new-onset AF at 1 year is higher in patients in which one of these vessels is stenosed at least 50% [56].

It has also been proved that coronary ischemia (either chronic or acute) may lead to changes in the structure and function of gap junction proteins, such as connexin 40 and 43, the conduction of action potential being slowed and nonhomogeneous. In this way, it is creates a substrate for the AF reentry mechanism, which is also favored by the ischemic necrosis of cardiomyocytes and their replacement with fibrous tissue. Then, the proinflammatory status associated with coronary ischemia, expressed by high levels of cytokines, such as IL-6, will accelerate fibrosis in atrial cardiomyocytes by modulating MMP2 (matrix metalloproteinase-2) expression [57,58,59].

The complications of coronary ischemia such as heart failure, diastolic dysfunction, and mitral regurgitation will lead to an atrial dilatation, which is an important key in the AF mechanism because as atrial cardiomyocytes stretch, they will be replaced with fibroblasts. These cells will accelerate collagen synthesis, which will favorize the genesis of reentry circuits. Moreover, atrial dilation may also affect cardiomyocyte electrical activity [60,61,62,63].

The focal ectopic activity in AF is sustained by coronary ischemia as a high level of intracellular calcium is the essential pathophysiological change in this disease. While some studies show that the central ischemic zone is the main ectopic source [64,65], others maintain that the peripheral area would be incriminated in the generation of ectopic foci [66].

Coronary ischemia is not only important in the pathogenesis of AF, but it also may play a role in its maintenance or even reappearance after catheter ablation. After several studies showed that clinical predictors such as enlarged left atrial diameter in echocardiography examination and the common risk factors (hypertension, obesity, type 2 diabetes mellitus, and dyslipidemia) have been shown to be a major factor in AF recurrence, a recent study, which involved 700 patients undergoing a strategy of pulmonary vein isolation for AF, showed that patients with coronary ischemia have also a higher recurrence of AF after ablation and, even more, the interventional treatment of coronary lesions may reduce this recurrence. After percutaneous coronary intervention, the E/E′ ratio had a value closer to the normal one and the diastolic dysfunction (which may be a mechanism of AF generation) was reduced, in this way explaining how the treatment of coronary lesions may prevent AF reappearance [67,68,69].

Regarding the patients who require coronary artery bypass graft (CABG) as treatment for CCS, it is well known that AF is one of the most frequent complications after this intervention, with an incidence between 30% and 50%. In addition to the classic risk factors (left atrial dilatation, age, hypertension, obesity, type 2 diabetes mellitus, dyslipidemia, and obstructive lung disease) for the development of postoperative AF, there is also oxidative stress, especially following ischemia caused by manipulation of the aortic cross clamp during surgery. Increased oxidative stress may lead to both electrical and structural remodeling of myocardial cells. On the one hand, it will cause alteration of desmosomes and gap junctions in cardiomyocytes. Thus, the conduction of the action potential will be impaired, which will shorten the refractory period, increasing the window in which an arrhythmia can take control of these cells. On the other hand, the mitochondrial function will be affected by the overexpression of mitochondrial caspases [70,71].

**Figure 3:**
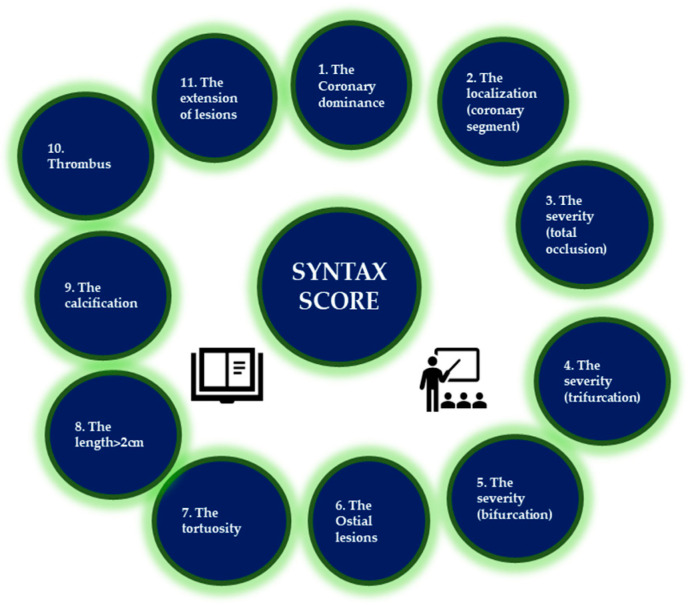
The 11 steps to assess the SYNTAX score.

HATCH score (Table 1) is used to estimate the risk of developing AF. A study shows that increasing the HATCH score from 0 to 7 increased the incidence of AF, and another claims its usefulness in predicting sinus rhythm metering after electrical cardioversion. SYNTAX score (Figure 3) is a unique tool to quantify the severity of coronary artery stenosis, particularly taking into account the number, the complexity, the severity, and the localization of stenosis [72,73].

There is an important correlation between HATCH and SYNTAX scores in assessing the risk of postoperative AF after CABG in patients with CAD. Further studies are needed to extend this correlation to patients with CCS, who do not require CABG, to determine their thromboembolic risk or even the risk of progression to AF [74].

Other scores capable of predicting AF recurrence after catheter ablation are R2CHADS2 (where R = renal dysfunction) and CHA2DS2-VASc [75]. Kornej et al. showed that a CHA2DS2-VASc ≥ 2 and a R2CHADS2 ≥ 2 are significantly associated with rhythm outcomes after catheter ablation [76]. Another score (MB-LATER score) may provide a more reliable predictive value for both late recurrence (after 3 months) and very late recurrence (after 1 year) after second-generation cryoballoon-based pulmonary vein isolation, according to Sano et al. [77]. Several other predictive scores for AF recurrence are cited in Table 2 [78,79,80,81,82,83,84,85].

### Chronic Coronary Disease as an Independent Factor for the Thromboembolic Risk in Patients with Atrial Fibrillation

In CHA2DS2-VASc score, peripheral arterial disease, considered ‘‘vascular disease’’, corresponds to one point. However, some studies have suggested different cardiovascular events in patients with coronary artery disease compared to those with peripheral arterial disease, including ischemic limb events, ischemic stroke, and cardiovascular death. In addition, one study reported poorer prognosis in patients with AF, peripheral arterial disease, and CCS compared to patients with CCS only or peripheral arterial disease only, and patients with AF and CCS had a higher risk of ischemic stroke than those with AF and peripheral arterial disease, particularly in those with a high CHA2DS2-VASC score [86].

Coronary ischemia is more than a promoter to sustain AF; it also represents an important independent risk factor for thromboembolic events in patients with AF. According to Steensig et al., the presence but not extent of CAD in the angiographic evaluation increased the number of thromboembolic events (ischemic stroke, transient ischemic attack, and systemic embolism) among patients with AF. This study is the reason why, in 2020, The European Society of Cardiology guidelines for the diagnosis and management of atrial fibrillation introduced “Angiographically significant CAD” in the point “V- Vascular Disease” of CHA2DS2-VASc score [6,87].

Two other variants of the CHA2DS2-VASc score, namely CHA2DS2-VASc-RAF (where R = renal dysfunction and AF = AF type) and CHA2DS2-VASc-LAF (where L = left atrial diameter and AF = AF type), have proven useful in estimating the risk of left atrial thrombus and spontaneous echo contrast in patients AF [88].

ATRIA Stroke Risk Score (history of stroke, age, sex category, history of diabetes mellitus, history of congestive heart failure, history of hypertension, proteinuria and eGFR< 45 or end-stage renal disease) was validated successfully in performing the prediction of thromboembolic events in patients with AF and it should improve the antithrombotic decision for patients with AF [89].

Assessment of the risk of death from thromboembolism in the elderly population with chronic coronary syndrome and atrial fibrillation is important for prevention given that, by 2050, the overall proportion of people over 65 years is projected to reach nearly 16%, and by 2100 nearly 23%, according to World Population Prospects [90].

## 5. From Atrial Fibrillation to Coronary Ischemia

AF may promote or even generate coronary ischemia by increased heart rate and thus increased oxygen consumption by accelerating atherosclerosis and by its high prothrombotic status following the loss of atrial systole. According to a study, patients with AF more frequently suffer from CCS with stenosis in the coronary tree ≥ 50%, in which the left main or proximal left anterior descending artery are usually implicated [8,91].

On the one hand, AF accelerates atherosclerosis by promoting endothelial dysfunction; it increases the serum level of coagulation factor VIII and it disrupts the release of nitric oxide. A study showed high levels of circulating endothelial cells, suggesting an endothelial dysfunction, in patients with AF [37,38].

On the other hand, AF is associated with systemic inflammation, which will promote atherosclerosis. A study claimed that the Stuart–Prower factor is able to activate the FX-PAR2 pathway, which will lead to the activation of macrophages, creating a proinflammatory status, that can accelerate the evolution of the plaque to an unstable one [39,40].

Then, AF may generate a mismatch between oxygen supply and demand by increasing the heart rate and by irregular and rapid contractions of the atrium. Moreover, in patients with AF, resistance in the coronary artery is affected in favor of the loss of coronary vasodilatation and implicitly the decrease in blood flow in these vessels [41,42]. Last but not least, although in rare cases, AF can be the cause of an acute cardiac ischemia by embolic mechanism [43].

AF may also generate thrombosis in the stable plaque by increased platelet activity and by the activation of coagulation system. In patients with AF, Choudhury et al. showed a greater activity of platelet surface factors CD62P and CD63, which stimulate the platelet adhesion to the plaque [44].

### CHA2DS2-VASc Score and Its Derivatives as a Predictive Factor for the Severity of Coronary Stenosis

According to Wojszel et al., a CHA2DS2-VASc score ≥ 4 may be a useful predictor for selecting patients to undergo coronary angiographic exploration for the diagnosis of CCS. Furthermore, another study claims that the CHA2DS2-VASc score may be an independent predictor for SYNTAX score, along with left ventricular ejection fraction, creatinine, C-reactive protein, serum LDL, and HDL cholesterol levels in patients with non-ST-segment elevation acute myocardial infarction (NSTEMI) and without AF. It seems that a CHA2DS2-VASc score > 4 implies a higher SYNTAX score and thus higher in-hospital mortality [92,93].

The Framingham Risk Score, which estimates the individual risk of a patient with CCS using classic risk factors (age, gender, systolic blood pressure, hypertension treatment, total cholesterol, HDL cholesterol, and smoking) is the most widely used, estimating the 10-year risk of developing myocardial infarction or death. However, this score cannot assess the severity of coronary artery disease, unlike the CHA2DS2-VASc-HSF score (which adds one point for hyperlipidemia, smoking, and family history of heart disease). This score is found to be extremely useful for assessing the severity of CCS according to recent data supporting the significant correlation between it and the Gensini coronary artery angiographic assessment score (Table 3) [94].

Two other variants of the CHA2DS2-VASc score, namely CHA2DS2-VASc-RAF (R assigns 1 point for renal dysfunction and AF is the type of AF) and CHA2DS2-VASc-LAF (L is left atrial diameter and AF is the type of AF), have been shown to be very useful in the prediction of left atrial thrombosis and spontaneous contrast on echocardiographic examination in patients with nonvalvular AF, but further studies are needed to establish any possible link also with the presence of CCS in these patients [88].

## 6. Atrial Fibrillation, Chronic Coronary Disease, and Their Common Risk Factors

Hypertension, smoking, obesity, type 2 diabetes mellitus, and dyslipidemia may represent an important risk factor for both AF and CCS [95].

Hypertension is the most common risk factors for both AF and CCS; it contributes to atrial dilatation by left ventricular hypertrophy and diastolic dysfunction. Moreover, atrial fibrosis is favorized by the increased expression of proinflammatory cytokines, caused by alterations in the renin angiotensin aldosterone system. Left ventricular hypertrophy will also increase oxygen consumption, generating a mismatch between oxygen supply and demand, promoting CCS [96].

Smoking accelerates atherosclerosis and it is a risk factor for atrial fibrillation as well. On the one hand, smoking induces oxidative stress, vascular inflammation, platelet coagulation, vascular dysfunction, and affects the serum lipid profile in both current and chronic smokers and active and passive smokers, which will accelerate atherosclerosis and will promote the development of CAD. On the other hand, smoking not only can predispose to atrial fibrillation through mediators such as chronic pulmonary obstruction/impaired lung function and hypertension, but it also may bring about a higher risk of thromboembolism or death in patients with AF [97,98].

In diabetic and obese patients, an increased mass of epicardial adipose tissue secretes an excess of profibrotic and proinflammatory adipokines, leading to the generation of intramyocardial fibrofatty infiltrates. On the one hand, diabetes is associated with chaotic energy use in the myocardium, which increases intramyocardial fat. Fibrofatty infiltrates are associated with abnormal calcium handling in cardiomyocytes and impaired repolarisation, promoting the generation of extrasystoles. In addition, the inflammatory phenotype determines connexin protein lateralization, which will affect the unidirectional conduction. These changes create a substrate for AF [9]. On the other hand, inflammatory adipokines in perivascular fat may modify intraluminal homeostasis through an “outside-to-inside” mechanism. Epicardial adipose tissue is a significant source of sympathetic neurotransmitters which can accelerate the diastolic dysfunction with eventual heart failure and the development of CCS. There is evidence that CCS is associated with a brown to white transition of epicardial fat, leading to an increased oxygen free radical production and accelerated coronary atherosclerosis [9].

The triglyceride glucose index is a surrogate of insulin resistance and may be calculated with the following mathematical formula: ln(fasting triglyceride[mg/dL] × fasting glucose [mg/dL]/2). A recent study, which involved 1953 hypertensive patients who underwent coronary computed tomography angiography, claimed a triglyceride glucose index used to predict the presence of CAD. Another study sustained that in patients already known with CCS, this index may be used as an important predictor for the progression of coronary lesions. In this way, triglyceride and serum glucose levels are directly linked to both the onset and progression of coronary ischemia [99,100].

Regarding dietary habits, it seems that alcohol consumption may be an important risk factor for AF, but on the contrary, moderate alcohol consumption has been associated with a lower risk of coronary artery disease, while Larson et al. showed risk ratios among drinkers of <12 g alcohol/drink in a cohort study with 7245 AF cases (this association could be explained by several mechanisms: shortening of right atrial effective refractory periods, changes in oxidative stress, vagal activity, and electrolyte imbalances), and Song et al. found a lower risk of CAD with light-to-moderate alcohol consumption. Ethanol may raise high-density lipoprotein cholesterol and apolipoprotein A-I; it may also influence atherosclerotic plaque composition and provide stabilizing benefits and improve insulin sensitivity which lowers the risk of type 2 diabetes [101,102].

Regarding the relationship with AF, these risk factors not only promote the appearance of this tachyarrhythmia but also contribute to its maintenance or recurrence. Correcting some of them, such as weight loss, has been associated with the success of treatment for maintaining cardiac sinus rhythm [103].

Metabolic-associated steatohepatitis often coexists in these patients. It can be determined by using fatty liver index (FLI) score values (based on body mass index, waist circumference, triglyceride, and gamma–glutamyl transferase levels) and it is a strong and independent predictor of a calcium score of 100 AU or higher in asymptomatic patients. It is associated with an increased risk of developing CCS in addition to traditional risk factors. However, a recent study has associated metabolic steatohepatitis with an increased risk of major adverse cardiovascular events, independent of other cardiovascular risk factors or the extent of coronary lesions [104].

## 7. The Vicious Circle between AF and CCS

**Figure 4:**
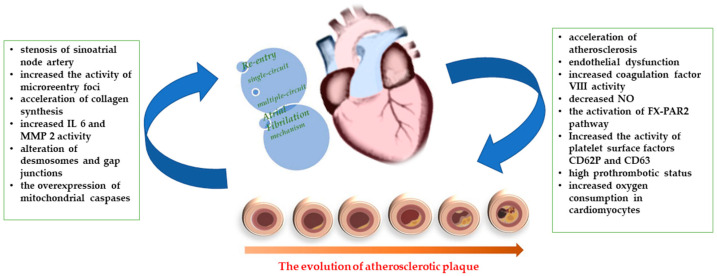
The vicious circle between atrial fibrillation and chronic coronary syndrome.

The association between CCS and AF is common, creating a complex clinical condition in which chronic oral anticoagulation is required when the CHA2DS2-VASc score is at least two. Each pathology leads to the promotion of the other through numerous mechanisms, which are summarized in Figure 4, and they share many common risk factors, such as hypertension, diabetes mellitus, dyslipidemia, obesity, and obstructive sleep apnea [3].

There are many studies that claim the influence of these two pathologies on each other. In Table 4 and Table 5, some of the most important studies and the pathophysiological mechanism behind each are mentioned.

### Destroying the Circle—Antithrombotic Therapy in Patients with Atrial Fibrillation and Chronic Coronary Syndrome

Chronic oral anticoagulation reduces the risk of embolism but also increases the risk of bleeding, especially in elderly, frail patients. Thus, these types of patients requiring anticoagulation for AF have a higher risk of adverse effects, but they also receive an absolute benefit from chronic oral anticoagulation. However, there are data suggesting that chronic oral anticoagulation in elderly patients with CCS and AF is not sufficient to protect against thromboembolic death, which could be attributed to an increased risk of death in patients with a higher CHA2DS2-VASc score [105].

In antithrombotic therapy for patients with CCS and AF, in addition to chronic oral anticoagulation, it is vital to add the antiplatelet (clopidogrel in most cases to avoid a high bleeding risk) or even double antiplatelet (both acetylsalicylic acid and a P2Y12 inhibitor) for patients treated by percutaneous coronary intervention to reduce stent thrombosis and the risk of cardiovascular death. It is important in each case to balance the thrombotic risk and risk of bleeding in choosing the optimal duration of triple therapy (one week versus one month). One year after revascularization therapy, anticoagulant therapy alone appears to be the most beneficial choice for the patient in terms of bleeding risk compared to the combination of an anticoagulant and an antiplatelet agent [3,106]. AFIRE trial also confirms the noninferiority of rivaroxaban monotherapy versus the dual therapy (combination of rivaroxaban and a single antiplatelet agent) in patients with atrial fibrillation and stable coronary artery disease/patients with atrial fibrillation and who had percutaneous coronary intervention (PCI) or coronary-artery bypass grafting (CABG) more than 1 year earlier. In addition, rivaroxaban monotherapy was superior in terms of safety regarding the bleeding risk in these patients [107]. Another recent meta-analysis claims also that in patients with AF and CCS, anticoagulant + single antiplatelet agent dual therapy vs. anticoagulant monotherapy is associated with a statistically significant increase in major bleeding, and there is no significant reduction in thrombotic events, cardiovascular mortality, and all-cause mortality [108].

Since non-vitamin K anticoagulants (NOACs), including dabigatran, apixaban, rivaroxaban, and edoxaban, are available, they are preferred over acenocoumarol and warfarin in cerebrovascular and systemic thrombo-embolic event prevention for patients with non-valvular AF [109,110,111,112]. AUGUSTUS trial showed that for people with AF and coronary ischemia, apixaban and a P2Y12 inhibitor should be used at the expense of warfarin or dual antiplatelet agents [113,114].

Other therapeutic strategies include the use of statins and lipid-lowering drugs to stabilize cholesterol plaques. It has been proved that statin could reduce the incidence of CCS in patients with AF and vice versa. For coronary ischemia, revascularization therapy by percutaneous coronary intervention or coronary artery bypass graft is essential for patients’ prognosis and, moreover, it may reduce AF onset. For people with AF, conversion to sinus rhythm using antiarrhythmic drugs, electrical cardioversion, or catheter ablation is vital and will reduce oxygen consumption, preventing the evolution of coronary ischemia. If AF is declared permanent, rhythm control with beta-blockers is essential in preventing the occurrence of myocardial ischemia [115,116,117].

Regarding anti-arrhythmic therapy, it is well known that class 1C antiarrhythmic drugs are effective first-line agents for AF treatment, but these agents, especially encainide and flecainide, are commonly avoided in patients with known coronary artery disease (especially postmyocardial infarction) because they may increase mortality in this case. However, Pantlin et al. showed that (in a study with only 78 patients) treatment with 1C antiarrhythmic drugs appears not to increase mortality in patients with AF, preserved left ventricle function, and occult CAD indicated by PET Stress Testing with Coronary Flow Capacity. Another study showed also that in patients with mild to moderate CAD, using propafenone after AF ablation may have a safety profile similar to amiodarone, and, most importantly, it is not associated with major arrhythmic events [118,119,120].

Besides amiodarone, another class III antiarrhythmic, dofetilide, may be used in patients with AF and heart failure or coronary artery disease. The effectiveness of dofetilide was established for AF rhythm control in patients with a history of coronary artery disease in a cohort of 287 patients [121].

Last but not least, and by far the most important, is the primary prevention strategy of controlling risk factors such as hypertension, dyslipidemia, obesity, blood glucose, and lifestyle (smoking, diet, and physical activity) to avoid the installation of coronary ischemia or AF and the creation of the vicious circle between the two of them [122].

## 8. Conclusions

Atrial fibrillation, the most common arrhythmia and chronic coronary syndrome, of one of the forms of coronary ischemia, often coexist as they share many common risk factors. They have a huge influence on each other, promoting and aggravating each other through pathophysiological mechanisms, leading to a challenging vicious circle.

In the presence of comorbidities associated with chronic coronary syndrome and atrial fibrillation, especially their common risk factors (hypertension, obesity, type 2 diabetes mellitus, smoking, dyslipidemia, and metabolic-associated steatohepatitis), the risk of thromboembolic complications and death increases further by age.

## Figures and Tables

**Table 1:** HATCH score for predicting the onset of atrial fibrillation.

Parameter	Points
H (Hypertension)	1p
A (Advanced age > 75 years)	1p
T (TIA/TIS)	2p
C (COPD)	1p
H (HFrEF)	2p

AF—atrial fibrillation; TIA—transient ischemic attack; TIS—transient ischemic stroke; COPD—chronic obstructive pulmonary disease; HFrEF—heart failure with reduced ejection fraction.

**Table 2:** Predictive scores for AF onset/recurrence.

Reference	Predictive Scores for AF Onset/Recurrence
Emren et al., 2016 [72]; Suenari et al., 2017 [73].	HATCH
Lohrmann et al., 2022 [75]; Kornej et al., 2014 [76].	CHA2DS2-VASc
Kornej et al., 2014 [76].	R2CHADS2
Sano et al., 2020 [77]; Mujović N et al., 2017 [78].	MB-LATER
Mesquita J. et al., 2018 [79].	ATLAS
Canpolat U. et al., 2013 [80].	BASE-AF
Heeger C.H. et al., 2018 [81]; Akkaya E. et al., 2018 [82].	PLAAF
Winkle R.A. et al., 2016 [83].	CAAP-AF
Kosiuk J. et al.,2015 [84].	DR-FLASH
Kornej et al., 2015 [85].	APPLE

**Table 3:** Gensini score.

Reduction in Lumen Diameter	Severity Score—Points
1–25%	1p
26–50%	2p
51–75%	3p
75–90%	3p
91–99%	16p
100%	32p

**Table 4:** The pathophysiological mechanisms in trials from coronary ischemia to atrial fibrillation.

Trial	Pathophysiological Mechanism
Pokorney et al., 2022 [56].	Stenosis of sinoatrial node artery
Shu, 2017 [58].	Alteration of desmosomes and gap junctions (connexin 40 and 43) and increased IL6 and MMP 2 activity in atrial cardiomyocytes
Bahouth et al., 2010 [60].	Acceleration of collagen synthesis in atrial cardiomyocytes and the genesis of reentry circuits
Kornej et al., 2015 [68]; Hiraya et al., 2019 [69].	Diastolic dysfunction
Oktay et al., 2014 [70].	Overexpression of mitochondrial caspases in atrial cardiomyocytes

**Table 5:** The pathophysiological mechanisms in trials from atrial fibrillation to coronary ischemia.

Trial	Pathophysiological Mechanism
Bocchino et al., 2021 [3].	Increased oxygen consumption in cardiomyocytes
Liang et al., 2021 [8].	Stenosis in the coronary tree ≥ 50% (the left main or proximal left anterior descending artery are usually implicated)
Wojszel et al., 2022 [93].	Endothelial dysfunction, Increased coagulation factor VIII activity and decreased nitric oxide
Zheng et al., 2022 [88]; Andrianto et al., 2020 [94].	Systemic inflammation, the activation of macrophages by FX-PAR2 pathway,
Won et al., 2020 [100].	Stenosis in the coronary tree by embolic mechanism

## Data Availability

The data is contained within this article.
